# Strategy for efficient generation of numerous full-length cDNA clones of classical swine fever virus for haplotyping

**DOI:** 10.1186/s12864-018-4971-8

**Published:** 2018-08-09

**Authors:** Camille Melissa Johnston, Ulrik Fahnøe, Graham J. Belsham, Thomas Bruun Rasmussen

**Affiliations:** 1 DTU National Veterinary Institute, Technical University of Denmark, Lindholm, DK-4771 Kalvehave, Denmark; 2 Copenhagen Hepatitis C Program (CO-HEP), Department of Infectious Diseases, Hvidovre Hospital, Hvidovre, Denmark; 3 Department of Immunology and Microbiology, Faculty of Health and Medical Sciences, University of Copenhagen, Copenhagen, Denmark

**Keywords:** RNA, Genome, Bacterial artificial chromosome, RNA virus, Pestivirus, Haplotyping

## Abstract

**Background:**

Direct molecular cloning of full-length cDNAs derived from viral RNA is an approach to identify the individual viral genomes within a virus population. This enables characterization of distinct viral haplotypes present during infection.

**Results:**

In this study, we recover individual genomes of classical swine fever virus (CSFV), present in a pig infected with vKos that was rescued from a cDNA clone corresponding to the highly virulent CSFV Koslov strain. Full-length cDNA amplicons (ca. 12.3 kb) were made by long RT-PCR, using RNA extracted from serum, and inserted directly into a cloning vector prior to detailed characterization of the individual viral genome sequences. The amplicons used for cloning were deep sequenced, which revealed low level sequence variation (< 5%) scattered across the genome consistent with the clone-derived origin of vKos. Numerous full-length cDNA clones were generated using these amplicons and full-genome sequencing of individual cDNA clones revealed insights into the virus diversity and the haplotypes present during infection. Most cDNA clones were unique, containing several single-nucleotide polymorphisms, and phylogenetic reconstruction revealed a low degree of order.

**Conclusions:**

This optimized methodology enables highly efficient construction of full-length cDNA clones corresponding to individual viral genomes present within RNA virus populations.

**Electronic supplementary material:**

The online version of this article (10.1186/s12864-018-4971-8) contains supplementary material, which is available to authorized users.

## Background

Classical swine fever (CSF) is a highly contagious disease of pigs caused by infection with classical swine fever virus (CSFV) which belongs to the genus *Pestivirus*, within the *Flaviviridae* family. Pestiviruses are enveloped and the particles contain a linear, positive-sense RNA of approximately 12.3 kb. This genome includes a single, long, open reading frame (ORF) encoding a large polyprotein, flanked by 5′ and 3′ untranslated regions (UTRs) [1] that are critical for the autonomous replication of the genome [2, 3]. The viral polyprotein is co- and post-translationally processed by cellular and viral proteases to yield 12 mature products. There are 4 structural proteins (C, E^rns^, E1 and E2) and 8 non-structural proteins (N^pro^, p7, NS2, NS3, NS4A, NS4B, NS5A, and NS5B) [1]. Positive-strand RNA viruses evolve rapidly, due to error-prone RNA replication and the lack of proof-reading activity of the RNA-dependent RNA polymerase [4]. The high error rate results in a virus population that exists as a quasispecies (different, but closely related variants). These variants form a flat fitness landscape in sequence space of a selectively neutral network of variants, making the population more robust to withstand mutations and evade host responses [5]. Within this sequence space, certain variants, or haplotypes, may exist either with single nucleotide (nt) changes or, alternatively, predominantly in combination with other changes within the same genome. The diversity and quasispecies composition of CSFV and other pestiviruses have not been studied in great depth. Limited analyses of the evolutionary forces that drive sequence change, and the role of the quasispecies composition as a determinant of virulence have been reported [6, 7]. Consensus sequencing (and even deep sequencing) cannot easily resolve the different haplotypes that constitute the whole population. Obtaining full-length cDNA clones represents an approach to identify the individual haplotypes present within the virus population and also enables phenotyping. However, a prerequisite for this is generation of full-length cDNA suitable for cloning.

In the present study, the generation of full-length cDNA clones was achieved by the use of long RT-PCR for full-length genome amplification in combination with TOPO XL-2 and In-Fusion cloning. Numerous full-length cDNA clones representing the diversity within the CSFV population were obtained directly from RNA present within the serum of virus-infected pigs. This methodology provides the necessary tools for the robust characterization of virus subpopulations and haplotypes.

## Methods

### Primers

Oligonucleotide primers used are listed in Additional file 1.

### Preparation of full-length cDNAs from viral RNA

Viral RNA was extracted, using a combined TRIzol/RNeasy protocol [8] from a serum sample collected at 7 days post-inoculation (dpi) from a euthanized (by intravascular injection of pentobarbital) crossbred pig obtained from the high health status swine herd at DTU. The pig had been infected with vKos (rescued from the BAC clone Kos (GenBank KF977607.1, [9]) and passaged once in PK15 cells) and exhibited severe clinical signs of CSFV infection. This extracted RNA was used to generate full-length cDNA amplicons, using a modified version of the long RT-PCR method described previously [9, 10]. Briefly, the total RNA was reverse transcribed using the Maxima H Minus Reverse Transcriptase (Thermo Scientific) and the specific cDNA primer, CSF-cDNA-1 (Additional file 1). The cDNA was then amplified by long PCR using the primers CSF-Kos_1–59 and CSF-Kos_12313aR (Additional file 1) and the Q5 high-fidelity DNA polymerase kit (New England BioLabs). The products were gel purified with a GeneJET Gel extraction kit (Thermo Scientific) and quantified using a Qubit Fluorometric quantitation system (Thermo Scientific).

### Production of bacterial artificial chromosomes (BACs)

The cDNA amplicons were inserted into the BAC vector pBeloBAC11 (New England BioLabs) using the In-Fusion HD Eco-Dry Cloning Kit (Clontech). Briefly, pBeloBAC11 was converted to a linearized form using the long PCR as above, with primers Kos15_NotI_pBelF and Kos15_NotI_pBelR (Additional file 1), which contain 15-nt overhangs identical to regions of the primers used for the preparation of the cDNA amplicons. The linear product was gel purified and quantified as described above. The linearized pBeloBAC11 vector was mixed with amplicons (ca. 1:2 molar ratio) in a volume of 10 μl, which was transferred to the In-Fusion HD EcoDry tube. The mixture was incubated at 37 °C for 15 min and then at 50 °C for 15 min. A 2.5 μl aliquot was used to transform *E. coli* Stellar competent cells according to the manufacturer’s protocol, and colonies grown on LB plates containing chloramphenicol (CAM) (15 μg/ml).

### Cloning of amplicons in pCR XL-2-TOPO vector

The cDNA amplicons were also cloned using the TOPO XL-2 kit (Thermo Scientific). Briefly, the linearized pCR-XL-2-TOPO vector was mixed with amplicons (ca. 1:2 molar ratio) in a volume of 6 μl, according to the manufacturer’s protocol. pCR-Xl-2-TOPO is a linearized vector covalently bound at the 3′ ends of each DNA strand to the vaccinia virus DNA topoisomerase I [11]. The mixtures of vector and amplicons were incubated at 25 °C for 30 min. A 2-μl aliquot was used to transform *E. coli* One Shot™ OmniMAX™ 2TI^R^ competent cells according to the manufacturer’s protocol, and colonies were grown on LB plates containing kanamycin (KAN) (50 μg/ml) and 1 mM isopropyl β-D-1-thiogalactopyranoside (IPTG).

### Characterization of cloned cDNAs

BACs containing cDNA inserts were identified by colony PCR targeting the vector-insert junction at the terminus of the CSFV 5’ UTR, using primers CSF192-R and pBelo69R (Additional file 1) and visualized on 1% TAE agarose gels. Selected BACs and TOPO X-2 clones were purified from 4 ml overnight (ON) cultures (LB medium with 12.5 μg/ml CAM and 50 μg/ml KAN, respectively) using the GeneJET Plasmid Miniprep kit (Thermo Scientific), and the presence of full-length CSFV cDNA inserts was identified using *Not*I digestion and visualized on 0.5% TAE agarose gels. The presence of full-length CSFV cDNA was confirmed by long PCR and full-length sequencing, as described previously [9, 12].

### Validation of fidelity of cloning systems

Cloned cDNA amplicons were used for assessing the fidelity of the PCR and cloning process. For this, full-length amplicons were obtained from a cloned BAC cDNA (termed BAC clone Kos_KSP) corresponding to a variant of the CSFV strain Koslov [7], which contains 2 non-synonymous mutations in the coding region for the E2 glycoprotein (resulting in the E72K and L75P substitutions). Briefly, the cloned BAC cDNA was used as the template in a long PCR with the primers CSF-Kos_1–59 and CSF-Kos_12313aR (Additional file 1) and the Q5 high-fidelity DNA polymerase kit (New England BioLabs). The products were digested with *Dpn*I (Fast Digest, Thermo Scientific), gel purified with a GeneJET Gel extraction kit (Thermo Scientific) and quantified using a Qubit Fluorometric quantitation system (Thermo Scientific). In-Fusion and TOPO XL-2 cloning was performed as described above. The resultant In-Fusion clones were screened by colony PCR and differentiated from the original clone using *Sma*I digestion of the vector-insert PCR product. The original Kos_KSP clone contains a T7 promoter and a *Sma*I site before the CSFV genome whereas the expected product lacks this site. Confirmation of correct insert size was performed as described above, on selected In-Fusion and TOPO X-2 clones. Selected full-length clones were then sequenced and compared to the original clone Kos_KSP, to determine the fidelity of the system.

### Next generation sequencing (NGS) and phylogenetic analysis

Full-length amplified cDNA amplicons and cDNA clones were sequenced by NGS at the DTU Multi-Assay Core (DMAC, Kgs. Lyngby, Denmark) using an Ion PGM system (Life Technologies, Carlsbad, USA). Consensus sequences were obtained by mapping the reads to the vKos reference sequence (KF977607.1) using CLC Genomics Workbench v.9.5.2 (CLC bio, Aarhus, Denmark). Consensus sequences were aligned using MAFFT in Geneious (Biomatters, Auckland, New Zealand). Low frequency single nucleotide polymorphisms (SNPs, > 0.5%) were identified for cDNA amplicons using a combination of BWA, Samtools, Lo-Freq-snp-caller, and SnpEff, as described previously [9, 12]. Phylogeny was constructed using MrBayes v3.2.1 [13, 14] on a full-length cDNA sequence alignment using the General Time Reversible (GTR) model with default parameters (nst = 6). The Markov chain Monte Carlo algorithm was run for 10,000,000 iterations, with a sampling frequency of 7000 using two independent runs with three chains each, in order to check for convergence. Burn in was set at 25% of samples. The consensus tree was visualized in FigTree v.1.4.3.

## Results

### Fidelity of PCR and cloning

**Fig. 1:**
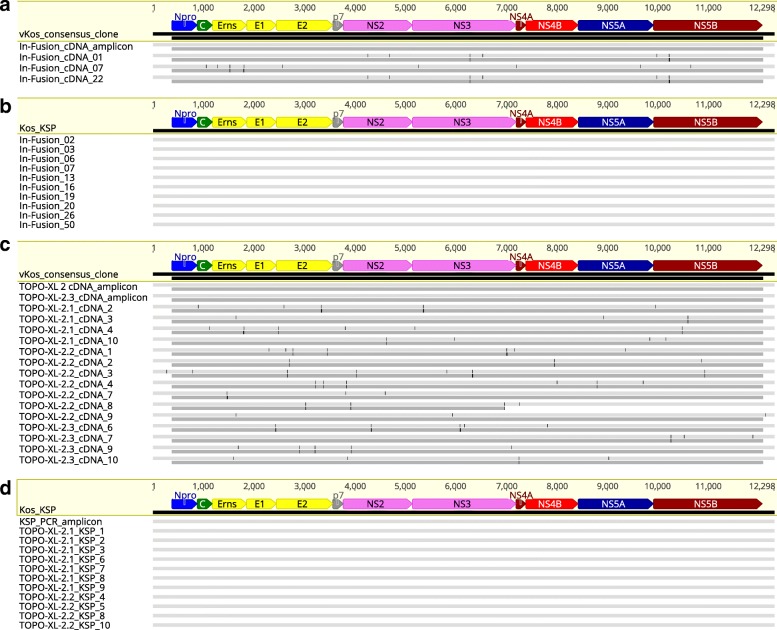
Multiple-sequence alignment of amplicons and cDNA clones. Multiple-sequence alignment of cDNA amplicons and cDNA clones (**a** and **c**) and Kos_KSP amplicons and clones (**b** and **d**). **a** In-Fusion cloning of cDNA amplicons generated from the serum of an infected pig. The cDNA amplicons used for the cloning is shown as the first sequence. **b** In-Fusion cloning of Kos_KSP derived amplicons. **c** TOPO-XL-2 cloning of cDNA generated from the serum of an infected pig. The sequences of two independent cDNA amplicons used for the cloning are shown **d** TOPO-XL-2 cloning of Kos_KSP derived amplicons. In **a** and **c** the top bars (light gray) depict differences from the consensus sequence at the nucleotide level, while the lower bars (dark gray) depict differences from the consensus sequence at the amino acid level

The fidelity of the PCR and cloning system was determined using the BAC clone Kos_KSP as the template for PCR and In-Fusion cloning of the generated amplicons (Table 1). In-Fusion cloning generated over 100 clones from each product and up to 50% of the screened clones were of the correct size. Ten randomly selected clones were full-length sequenced and the sequences were aligned with that of the original Kos_KSP, and showed 100% identity (Fig. 1). This shows that the long PCR together with the In-Fusion cloning process generates clones with high fidelity. The use of gel extraction to purify the amplicons, generated up to twice as many clones and 100% of the screened clones were of the correct size (Table 1).

**Table 1:** Efficiency of cloning full-length amplicons from Kos_KSP and cDNAs

Amplicon	Cloning method	# of clones	Positive/no. screened	#Correct insert size/#Screened with *Not*I	% correct of screened total	No. of clones sent for NGS
Kos_KSP	In-Fusion^d^	232	12/24	12/12	50%	5
Kos_KSP	In-Fusion^d^	111	8/24	8/8	33.3%	5
Kos_KSP	In-Fusion^b^	472	24/24	6/6	100%	–
Kos_KSP	In-Fusion liq.^b^	> 1000	10/10	10/10	100%	–
cDNA^a^	In-Fusion^b^	24	6/24	3/6	12.5%	3
Kos_KSP	TOPO XL-2^b^	> 1000	n.d.	8/10	80%	7
Kos_KSP	TOPO XL-2^b^	> 1000	n.d.	6/10	60%	4
cDNA^ac^	TOPO XL-2^b^	31	n.d.	4/10	40%	4
cDNA^ac^	TOPO XL-2^b^	43	n.d.	7/10	70%	7
cDNA^a^	TOPO XL-2^b^	33	n.d.	4/10	40%	4

*n.d.* Not done

^a^RNA derived cDNA

^b^Gel purified amplicons

^c^Same RT-PCR used

^d^amplicons purified by PCR clean-up kit

The fidelity of the cloning system using TOPO-XL-2 was also validated, as described for the BAC clones (see Table 1). TOPO XL-2 cloning generated over 1000 clones and up to 80% of the screened clones were of the correct size. Eleven of these clones were full-length sequenced and aligned as above, revealing a 100% identity (Fig. 1). This shows that the long PCR together with TOPO XL-2 cloning also produces clones with high fidelity.

Full-length amplicons from the Kos_KSP BAC or cDNA derived from RNA were cloned into the BAC or pCRXL-2-TOPO vector using In-Fusion or TOPO-XL-2, respectively. In-Fusion clones were screened using colony PCR, and subsequently positive colonies were further screened following *Not*I digestion to confirm the correct size of insert. TOPO-XL-2 colonies were also screened by *Not*I digestion to confirm the correct insert size.

### Cloning of cDNAs

Full-length cDNAs were amplified from CSFV RNA, isolated from serum of infected pigs (at 7 dpi), by long RT-PCR. Optimal results were obtained by substituting the SuperScript III reverse transcriptase with Maxima H minus Reverse Transcriptase, and the Accuprime High Fidelity with the Q5 high-fidelity DNA polymerase, the latter produces blunt-ended PCR products. Using these conditions, high yields of full-length amplicons of approximately 12.3 kb from the viral RNA were obtained (Additional file 2).

The amplified cDNAs were inserted directly into the linearized pBeloBAC11 vector by in vitro recombination using the In-Fusion system. Inserts of the correct size were identified by colony PCR, followed by restriction enzyme digestion with *Not*I (data not shown) and long PCR (Additional file 3a). A 10-fold lower number of bacterial colonies was observed compared to the cloning of the Kos_KSP-derived amplicons, and only 3 (12.5%) of these colonies contained an insert of the correct size (Table 1). These cDNA clones were full-length sequenced and aligned to the parental sequence. Two clones were 100% identical to each other representing one haplotype, but contained 6 SNPs compared to the reference vKos sequence, whereas the third clone was unique and had 9 SNPs (Fig. 1a).

Due to the low percentage of full-length cDNA clones obtained using the In-Fusion procedure, we then used the TOPO XL-2 cloning method, which relies upon the activity of DNA topoisomerase I. The amplified genomes were inserted directly into the linearized pCR-Xl-2-TOPO vector using DNA topoisomerase I. Transformation of *E. coli* with the products from three independent insertions into the vector yielded 31, 43, and 33 colonies (an average of 36). Correct inserts were identified by digestion with *Not*I (data not shown) and full-length PCR amplification (Additional file 3b). Up to 70% of the colonies contained an insert of the correct size (Table 1). Fifteen of these cDNA clones were full-length sequenced and these sequences were aligned with the parental consensus sequence. Each of the cDNA clones was unique with 2–8 SNPs compared to the reference sequence (Fig. 1c). A total of 68 SNPs were observed. Some 51.5% of all observed SNPs in the coding sequence were synonymous mutations while 48.5% were non-synonymous, out of which one resulted in the introduction of a premature stop codon; 3% of all the SNPs were located in the 5’ UTR and 3’ UTR, which together constitute approximately 5% of the total genome.

### SNP analysis of cDNAs used for cloning

**Fig. 2:**
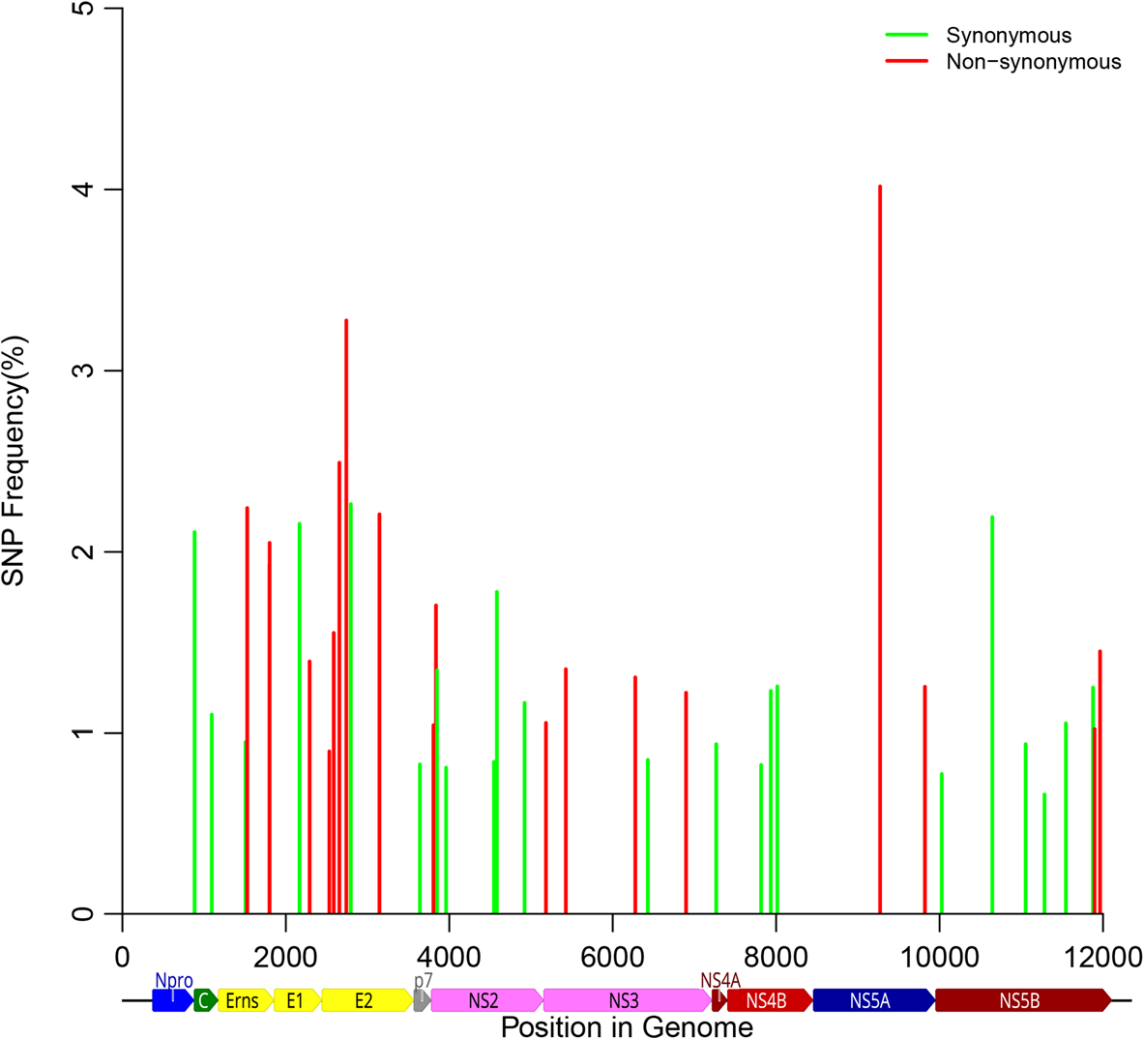
SNP analysis of RT-PCR amplicon. Deep sequencing of the amplicons derived by RT-PCR from the vKos infected serum at 7 days post infection. The histograms depict SNP frequency on the y-axis and genome position on the x-axis. The green and red colors indicate SNPs grouped as synonymous and non-synonymous, respectively

The cDNA amplicons derived from a serum sample, obtained from a CSFV Koslov-infected pig, were deep sequenced by NGS (coverage of approx. 11,000 reads per nt). The consensus sequence of these amplicons showed 100% identity to the vKos BAC clone. The cDNA amplicons used for the In-Fusion cloning displayed low-level sequence variation (below 5% frequency) scattered across the genome as synonymous and non-synonymous mutations (Fig. 2), which was also seen for the cDNA amplicons used for TOPO XL-2 cloning, although fewer SNPs were detected with a coverage of approx. 1800 and 2500 reads per nt, respectively. The SNPs (41) seen in the In-Fusion cDNA amplicons comprised both synonymous mutations (54%) and non-synonymous at 46%. Some 22% of the SNPs seen herein could also be detected in the cloned cDNAs, from both In-Fusion and TOPO-XL-2 cloning. A separate pool of amplicons, generated from a separate RNA extraction and RT-PCR of the same serum sample, was also deep sequenced by NGS (this produced a coverage of approximately 20,000 reads per nt). As above, the SNPs (693 in total) comprised both synonymous mutations (60.1%) and non-synonymous (39.9%) in the coding sequence with low-level sequence variation (below 5%) scattered across the genome, with 126 SNPs present at above 1% frequency. Some 50% of the SNPs observed in the cloned cDNAs could be detected in the amplicons sequenced at greater depth. An additional 20% of the SNPs detected in the cloned cDNAs could be detected in deep sequenced cDNA amplicons of serum from pigs infected with either vKos or vKos BAC clone variants (data not shown). Furthermore, another 9% SNPs have been observed in full-length CSFV genomes and full-length E2 glycoprotein sequences reported previously (complete coding sequence (CDS) and full-length E2 glycoprotein sequences were retrieved from GenBank [15] and the CSF database of the European Community Reference Laboratory [16] as described [17]). The unique mutations seen in the cDNA clones, most of which are located in the E2 and NS3 coding regions, are therefore most likely low frequency SNPs present in the populations (< 0.3%), and not errors introduced by the procedures (see Discussion).

### Phylogenetic reconstruction

**Fig. 3:**
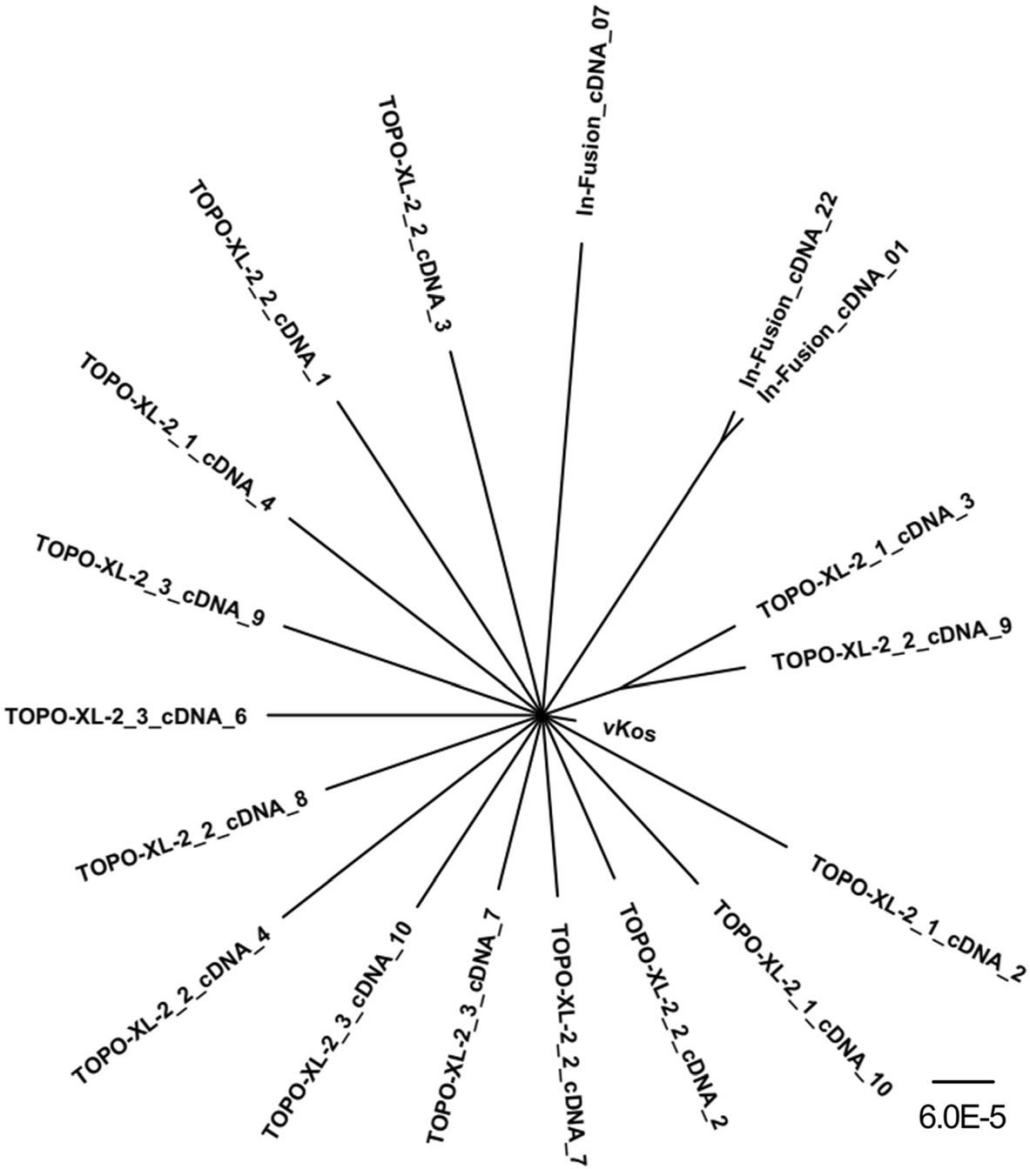
Phylogenetic reconstruction inferred from cDNA clones. Phylogenetic structure of the virus population present in the vKos infected pig serum inferred from the cDNA clones by tree reconstruction using MrBayes [14]

A phylogenetic tree for the cDNA clones was inferred using Bayesian methods (Fig. 3) in order to elucidate the population structure. The unrooted tree was star-like, with the cDNA sequences branching out from a single node that corresponds to the Koslov reference sequence. The majority of the cDNA clones were unique, forming no apparent clades, and with a low degree of order; just 2 In-Fusion clones were 100% identical and 2 TOPO XL-2 clones shared one mutation.

## Discussion

The aim of this study was to establish methodology for efficient, direct cloning of cDNAs corresponding to the full-length genomes of CSFV from infected pigs. Generation of full-length cDNA clones has often been a lengthy and arduous process. However, the technology is improving. BACs are ideally suited for the stable maintenance of large DNA/cDNA sequences derived from viral genomes [18]. Several BACs, containing full-length cDNAs of different CSFV strains, have been established [9, 12, 19]. Similarly, BACs containing cDNAs corresponding to the genomes of other members of the *Flaviviridae* family have also been described [10, 20–22]. In this study, In-Fusion cloning of amplicons derived from an established cDNA clone in a BAC vector was efficient, with an apparent fidelity of 100%. However, In-Fusion cloning of cDNA amplicons generated directly from viral RNA yielded only a few clones with the correct insert size. In contrast, using the TOPO XL-2 cloning method, we generated many more cDNA clones with a high proportion having the correct insert size. The advantages of this optimized methodology are: (i) circumvention of multiple cloning steps (since full-length amplicons can be directly inserted into plasmid vectors by in vitro recombination or using DNA topoisomerase), (ii) provision of a system for identifying sequence diversity within virus populations and the haplotypes within the entire genome, (iii) enabling the construction of genetically distinct viral cDNA clones and (iv) enabling phenotypic characterization of haplotypes.

The In-Fusion cloning system is not dependent upon the use of restriction enzymes or ligases and allows for the insertion of fragments at any suitable location within the vector [23]. In-Fusion cloning has previously been used for replacing cDNA corresponding to one CSFV strain with that of another in already established CSFV cDNA BAC clones [24, 25], and for CSFV field isolates using amplicons generated by in vitro overlap extension PCR [26]. CSFV BAC clones have previously been found to be stably maintained through multiple generations in *E. coli* [26], as seen for other full-length cDNA clones corresponding to other members of the *Flaviviridae* [19, 20, 22]. The stability of the TOPO XL-2 clones was not assessed here, as the constructs were used directly for NGS analysis, in order to map virus subpopulations and to perform haplotyping. However, if necessary, it should be possible to obtain a stable BAC clone, using the insert from a TOPO XL-2 clone, following PCR amplification which can then be cloned using the In-Fusion system since this cloned cDNA to amplicon cloning process works with high fidelity and success rate.

All cDNA clones generated from viral RNA contained SNPs, as expected, for individual virus genomes obtained from an RNA virus population, owing to its quasispecies nature. Interestingly, 3 of the SNPs detected in the TOPO XL-2 cDNA clones (G270A, T784C, and L764P) and one from the In-Fusion cDNA amplicon (A9821G) were also found at the consensus level in a contact pig infected with vKos at 6 dpi in the study by Fahnøe et al. [9]. In the present study, the SNP analysis of the cDNA amplicon used for In-Fusion cloning revealed the presence of 41 SNPs in total, all with a frequency below 5%, whereas the cDNA amplicon sequenced with 2-fold higher coverage, revealed 693 SNPs in total (< 5% frequency), with 126 SNPs detected above a frequency of 1%. In another study (Fahnøe U, Pedersen AG, Höper D, Beer M, Rasmussen TB., unpublished), we observed 159 SNPs scattered across the genome (> 1% frequency) in the inoculum used to challenge pigs; this inoculum was derived from the blood of pigs infected with the Koslov strain of CSFV [27]. Ten of these SNPs were observed at about 40% frequency. The larger number of SNPs found in the latter study at higher frequencies, is most likely due to the Koslov strain being a wild-type virus, which has had a lot of time to evolve in its natural host, whereas vKos, as used in the present study, is a virus preparation derived from a unique cloned cDNA. Therefore, the rescued virus may be expected to consist of a less diverse quasispecies with respect to high frequency haplotypes than a highly virulent “field” isolate, as reported previously [28]. Over 79% of the SNPs observed in the clones, were detected in either the RNA-derived amplicons used in our study, in the serum of pigs infected with either vKos or vKos BAC clone variants, or in published CSFV sequences. The remaining 21% of SNPs which were not observed elsewhere are most likely very low frequency SNPs in the viral population, and were located mostly in the E2 and NS3 coding regions. The N-terminal half of the E2 glycoprotein is one of the most variable regions in the CSFV genome [29], presumably due to E2 being under immune pressure. In addition, E^rns^ and NS3 also induce detectable antibodies [30–32]. E2 is under purifying selection pressure [33], likely due to the immune response, this is most probably also the case for NS3, although perhaps to a lesser degree, as it is a nonstructural protein and does not elicit neutralizing antibodies [34], however it is a strong activator of cytotoxic T lymphocytes [35–37]. In principle, these apparent SNPs could be due to errors by the reverse transcriptase; however, this is unlikely as most of the SNPs were detected in multiple independent cDNAs and also in previously published sequences. Furthermore, the SNPs which were not detected were randomly distributed, which one would not expect if these SNPs were caused by reverse transcriptase errors, due to the skewed nature of its error distribution [38]. However, as the virus population originally stems from a BAC clone, which was in vitro transcribed using a T7 RNA polymerase, errors could have been introduced into the virus inoculum by this process. This is not a concern when using field isolates, as no T7 RNA polymerase is utilized.

The phylogenetic reconstruction of the cDNA clones from both In-Fusion and TOPO XL-2 cloning formed no apparent clades, with a low degree of order. This is most likely due to the clone-derived virus inoculum used for the infection experiment, which is consistent with the less diverse quasispecies seen in the SNP analysis. This is also in agreement with our earlier study, in which a higher degree of order was seen in the phylogenetic reconstruction of cDNA clones from a field isolate of the moderately virulent CSFV strain Roesrath [12]. That SNP analysis also revealed several SNPs at a frequency above 5%; this is a typical quasispecies distribution, in which most haplotypes are present at a very low frequency while a few are dominant [7].

This optimized high-throughput strategy for obtaining full-length cDNA clones, described here, avoids the time-consuming and costly processes inherent in traditional methods. This methodology allows the identification of the individual components of the quasispecies that comprise the virus population and reveals how mutations are linked on individual viral genomes. This approach will help improve understanding of the molecular mechanisms involved in the virulence of CSFV, enabling new approaches for disease control including development of novel vaccine strategies.

## Conclusions

In summary, we report the high efficiency construction and sequence analysis of numerous full-length CSFV cDNA clones using RNA prepared directly from the serum of virus-infected pigs by applying long RT-PCR and TOPO XL-2 topoisomerase cloning. This methodology allows the identification of the individual haplotypes that constitute the viral quasispecies. In addition, it can be applied to any RNA virus for which the viral genome can be amplified by a long RT-PCR approach.

## Additional files

Additional file 1: List of oligonucleotide primers used in this study. (DOCX 17 kb)

Additional file 2: Gel electrophoresis of long RT-PCR amplicons used for In-Fusion and Topo XL-2 cloning. (DOCX 71 kb)

Additional file 3: Gel electrophoresis of full-length PCR products from generated cDNA clones. (DOCX 160 kb)

## Abbreviations

BAC: Bacterial artificial chromosome
CAM: Chloramphenicol
CDS: Coding sequence
CSF: Classical swine fever
CSFV: Classical swine fever virus
dpi: Days post-inoculation
GTR: General time reversible
IPTG: Isopropyl β-D-1-thiogalactopyranoside
KAN: Kanamycin
NGS: Next generation sequencing
nst: Number of substitution types
nt: Nucleotide
ON: Overnight
ORF: Open reading frame
SNP: Single nucleotide polymorphism
UTR: Untranslated region
