# Supplementary material for: Strategy for efficient generation of numerous full-length cDNA clones of classical swine fever virus for haplotyping
Source: BMC Genomics. 2018 Aug 9;19:600. doi: 10.1186/s12864-018-4971-8 (PMC6085635; doi:10.1186/s12864-018-4971-8)
Supplement: Supplementary file 1 — List of oligonucleotide primers used in this study. (DOCX 17 kb) [file 12864_2018_4971_MOESM1_ESM.docx]

**Additional file 1 – List of oligonucleotide primers used in this study.**

| **Primer** | **Sequence (5’**$\boldsymbol{\to}$**3’)** | **Reference** |
| --- | --- | --- |
| CSF-Kos_1-59 | GTA TAC GAG GTT AGT TCA TTC TCG TAT GCA TGA TTG GAC AAA TCA AAA TTT CAA TTT GG | Modified from [39] |
| CSF-Kos_12313aR | GGG CCG TTA GGA AAT TAC CTT AGT CCA ACT GT | [39] |
| CSF-cDNA-1 | GGG CCG TTA GGA AAT TAC CTT AG | This study |
| Kos15_NotI_pBelF | ATT TCC TAA CGG CCC GCG GCC GCA TCG AAT ATA ACT TCG T | This study |
| Kos15_NotI-pBelR | ACT AAC CTC GTA TAC GCG GCC GCC CGT CGA CCA ATT CTC ATG TTT GAC AGC TTA TC | This study |
| CSF192-R | CTA CTG ACG ACT GTC CTG TAC | [40] |
| pBelo69R | CGG ATG AAT GGC AGA AAT TCG ATG ATA AGC TGT CA | [41] |

39. Leifer I, Hoffmann B, Hoper D, Rasmussen TB, Blome S, Strebelow G, et al. Molecular epidemiology of current classical swine fever virus isolates of wild boar in Germany. J Gen Virol. 2010;91:2687–97.

40. Hoffmann B, Depner K, Schirrmeier H, Beer M. A universal heterologous internal control system for duplex real-time RT-PCR assays used in a detection system for pestiviruses. J Virol Methods. 2006;136:200–9.

41. Friis MB, Rasmussen TB, Belsham GJ. Modulation of translation initiation efficiency in classical swine fever virus. J Virol. 2012;86:8681–92.
