# Supplementary material for: Strategy for efficient generation of numerous full-length cDNA clones of classical swine fever virus for haplotyping
Source: BMC Genomics. 2018 Aug 9;19:600. doi: 10.1186/s12864-018-4971-8 (PMC6085635; doi:10.1186/s12864-018-4971-8)
Supplement: Supplementary file 2 — Gel electrophoresis of long RT-PCR amplicons used for In-Fusion and Topo XL-2 cloning. (DOCX 71 kb) [file 12864_2018_4971_MOESM2_ESM.docx]

**Additional file 2 – Gel electrophoresis of long RT-PCR amplicons used for In-Fusion and Topo XL-2 cloning.**


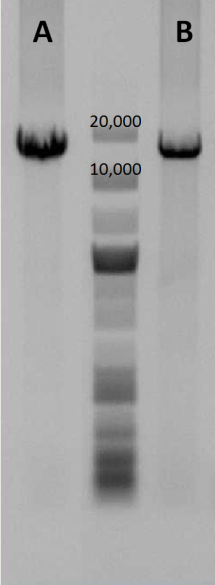


Full-genome amplification of CSFV RNA from serum sample by long RT-PCR and analyzed by agarose gel electrophoresis prior to In-Fusion (A) and TOPO-XL-2 (B) cloning.
