# Supplementary material for: Strategy for efficient generation of numerous full-length cDNA clones of classical swine fever virus for haplotyping
Source: BMC Genomics. 2018 Aug 9;19:600. doi: 10.1186/s12864-018-4971-8 (PMC6085635; doi:10.1186/s12864-018-4971-8)
Supplement: Supplementary file 3 — Gel electrophoresis of full-length PCR products from generated cDNA clones. (DOCX 160 kb) [file 12864_2018_4971_MOESM3_ESM.docx]

**Additional file 3 – Gel electrophoresis of full-length PCR products from generated cDNA clones.**

a)

b)


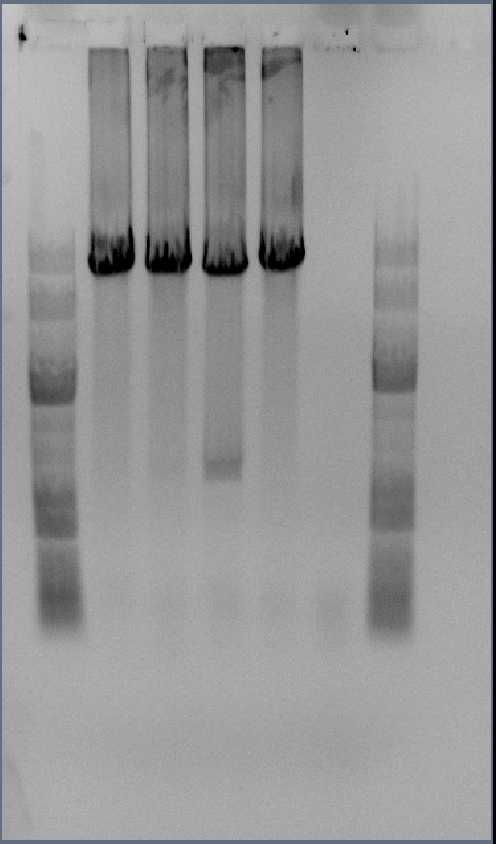


20,000

10,000

**1 7 22**


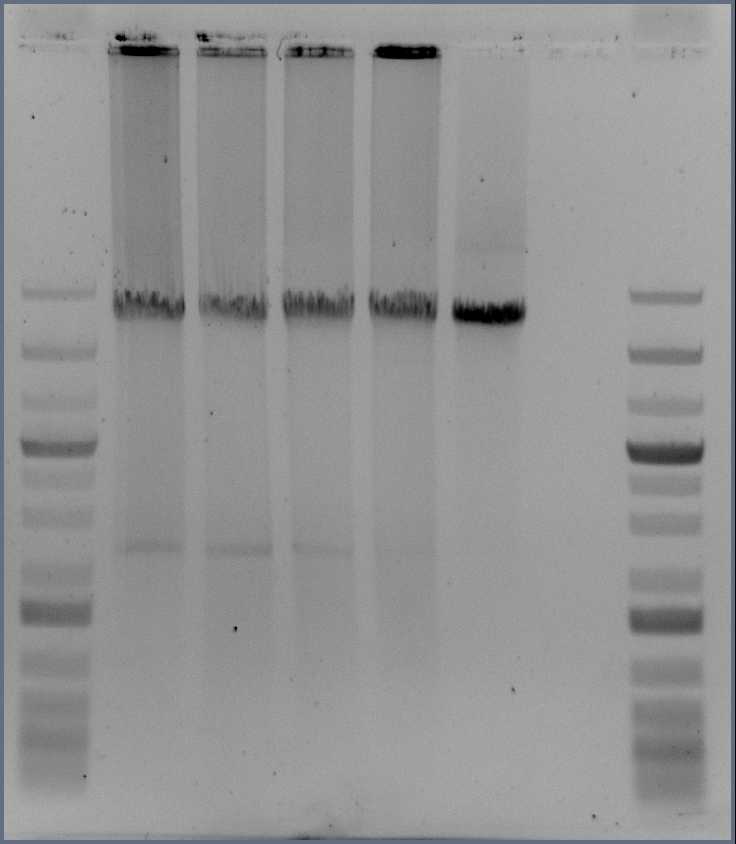


20,000

10,000

**2 3 4 10**

Full-genome amplification of generated cDNA from clones using long PCR. Correct sized clones were confirmed by full-genome amplification by long PCR and analyzed by agarose gel electrophoresis. a) BAC clones. b) TOPO XL-2 clones.
